# Wearable Sweat Rate Sensors for Human Thermal Comfort Monitoring

**DOI:** 10.1038/s41598-018-19239-8

**Published:** 2018-01-19

**Authors:** Jai Kyoung Sim, Sunghyun Yoon, Young-Ho Cho

**Affiliations:** 1 Department of Bio and Brain Engineering, Korea Advanced Institute of Science and Technology (KAIST), 271 Daehak-ro, Yuseong-gu, Daejeon 34141 Republic of Korea; 2 Present Address: Center for Medical Convergence Metrology, Korea Research Institute of Standards and Science (KRISS), 267 Gajeong-ro, Yuseong-gu, Daejeon 34113 Republic of Korea

## Abstract

We propose watch-type sweat rate sensors capable of automatic natural ventilation by integrating miniaturized thermo-pneumatic actuators, and experimentally verify their performances and applicability. Previous sensors using natural ventilation require manual ventilation process or high-power bulky thermo-pneumatic actuators to lift sweat rate detection chambers above skin for continuous measurement. The proposed watch-type sweat rate sensors reduce operation power by minimizing expansion fluid volume to 0.4 ml through heat circuit modeling. The proposed sensors reduce operation power to 12.8% and weight to 47.6% compared to previous portable sensors, operating for 4 hours at 6 V batteries. Human experiment for thermal comfort monitoring is performed by using the proposed sensors having sensitivity of 0.039 (pF/s)/(g/m^2^h) and linearity of 97.9% in human sweat rate range. Average sweat rate difference for each thermal status measured in three subjects shows (32.06 ± 27.19) g/m^2^h in thermal statuses including ‘comfortable’, ‘slightly warm’, ‘warm’, and ‘hot’. The proposed sensors thereby can discriminate and compare four stages of thermal status. Sweat rate measurement error of the proposed sensors is less than 10% under air velocity of 1.5 m/s corresponding to human walking speed. The proposed sensors are applicable for wearable and portable use, having potentials for daily thermal comfort monitoring applications.

## Introduction

Recently, the human thermal status monitoring has been required for human-machine interaction systems. For example, cognitive air conditioning systems^1^ use the human thermal status monitoring for controlling the surrounding temperature. Wearable devices such as smart jackets^2,3^ also require the human thermal status monitoring for checking physiological conditions.

There are three typical skin physiological signals^4–6^ representing the human thermal status: mean skin temperature, peripheral blood flow and sweat. Mean skin temperature^7,8^ is widely used for the human thermal status monitoring due to easy measurement. However, the mean skin temperature measurement requires a multiple number of the sensors attached on various spots of skin^9,10^, resulting in wearing irritation. The peripheral blood flow can be measured^11^ at the single peripheral spot of a human body such as fingertip, earlobe and toe. Despite the advantage of local measurement, the peripheral blood flow measurement is suffering from motion artifact^12^, thus requiring compensation process^13^. Several studies^14,15^ reported that amount of sweat is highly related to the thermal status. Sweat is stable to winds caused by human movement or external air flow and can be measured at the local body spot such as arm. The most widely used methods for sweat measurement are measuring skin conductance and sweat rate. Skin conductance measurement^16–18^ uses two electrodes which are attached on the skin and measure conductance changed by the electrolyte in sweat. The skin conductance measurement shows nonlinear characteristics due to the ion reabsorption phenomenon^19^. For this reason, the skin conductance is mainly used for psychophysiological studies^20^ using the narrow sweat range. Sweat rate^14^, meanwhile, measures the humidity evaporation rate depending on the sweat generation on skin. Sweat rate shows linear characteristics^15^ in the wide range of sweat, thus suitable for the human thermal status monitoring applications.

Sweat rate sensors are basically composed of two components: a humidity chamber for collecting the sweat-induced humidity on skin, and humidity sensors inside the chamber for measuring the collected humidity. Previous sweat rate sensors can be divided into forced ventilation methods and natural ventilation methods. The forced ventilation methods are composed of pump-based types and ice condenser-based types. The pump-based types^21,22^ measure the humidity difference between the air inlet and the air outlet of the chamber during the air circulation by pump while the ice condenser-based types^23–25^ use ice condenser freezing the humidity as a ventilation element. In general, the force ventilation methods having bulky sizes due to the external pumps and the air pneumatic systems or the ice condensor. The natural ventilation method, meanwhile, can be adapted as wearable prototypes due to their compact size characteristics. The natural ventilation methods are composed of open chamber types and closed chamber types. The open chamber types^26–30^, having two humidity sensors inside the chamber which end is opened, measure the humidity difference between the two humidity sensors positions while the generated sweat is naturally ventilated through the opened end. However, these types are strongly affected by the air flow^31^ even under the gentle wind of under 0.5 m/s, thus difficult to be used in mobile monitoring applications. The closed chamber types^32,33^, meanwhile, using a single humidity sensor inside the closed chamber measure the humidity rising rate while the chamber is closed by skin contact. Image analysis-based sensor^34^ uses time series photos of Macroduct instead of a humidity sensor for measuring the sweat flow rate. The closed chamber types have the portable size of under 200 g^32^ and show the stable measurement performance in the air flow conditions, thus having high potential for the mobile monitoring applications. The only weakness is that the chamber should be manually separated from skin after each measurement, in order for the chamber to ventilate collected humidity.

To solve the manual ventilation problem of the closed chamber types, we previously proposed the portable sweat rate sensor^35^ where the thermo-pneumatic actuator is integrated. The integrated thermo-pneumatic actuator lifts the closed chamber upon skin, thus successfully performing the automatic natural ventilation. The thermo-pneumatic actuator, however, has required the high operation power of over 12 W due to the large heat capacity of the expansion fluid used for actuation. The expansion fluid was large volume of 8.0 ml of ethanol having high boiling point of 78 °C. In this paper, to solve the high power problem, we propose new type of watch-type sweat rate sensor that optimally minimizes the expansion fluid volume to 0.4 ml through the thermal circuit-based estimation model. Also, the type of expansion fluid of the present watch-type sweat rate sensor is changed to PF-5060 with a lower boiling point of 56 °C. Therefore, the present watch-type sweat rate sensor can be operated with two commercial batteries (6 V) for about 4 hours while maintaining the sweat rate measurement performance comparable to the previous portable sweat rate sensors^35^. The present watch-type sweat rate sensor has the advantage of low-weight and low-power operation as well as wind-resistant characteristics, thereby highly applicable to daily human sweat rate monitoring applications such as the cognitive air-conditioning systems or the smart jackets.

## Results

### Principle

**Figure 1:**
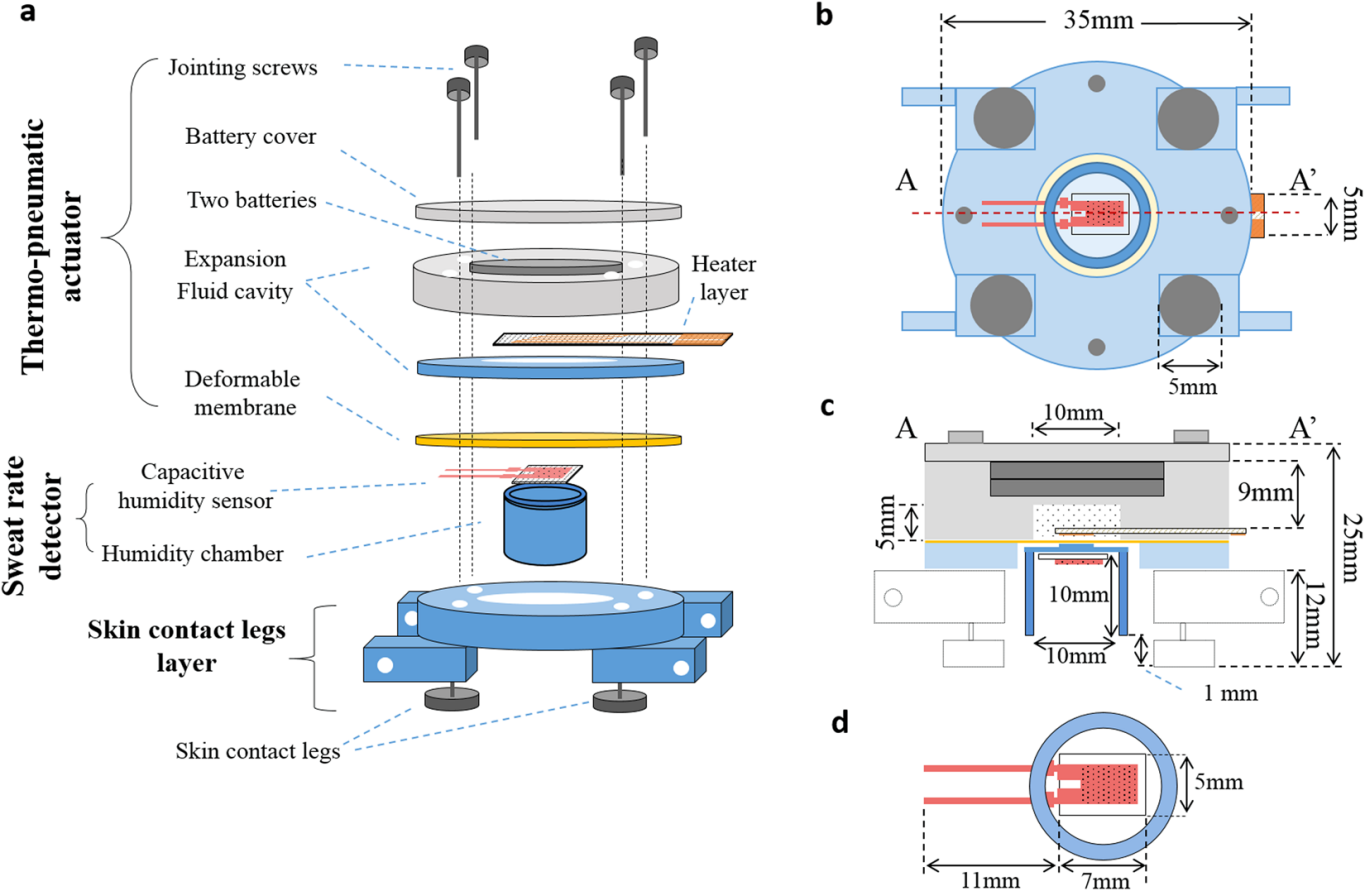
The watch-type sweat rate sensor: (**a**) overall view; (**b**) bottom view; (**c**) cross-sectional view along A-A’ in (**b**); (**d**) enlarged bottom view of the humidity chamber with capacitive humidity sensor.

Figure 1 shows the structure of the watch-type sweat rate sensor. The watch-type sweat rate sensor is composed of a thermo-pneumatic actuator, a humidity chamber, and a skin contact legs layer (see Fig. 1a). The thermo-pneumatic actuator moves the humidity chamber above skin. The humidity chamber includes a single capacitive humidity sensor inside it. The skin contact legs layer can hang wrist bands at both ends, capable to be watch-type device.

**Figure 2:**
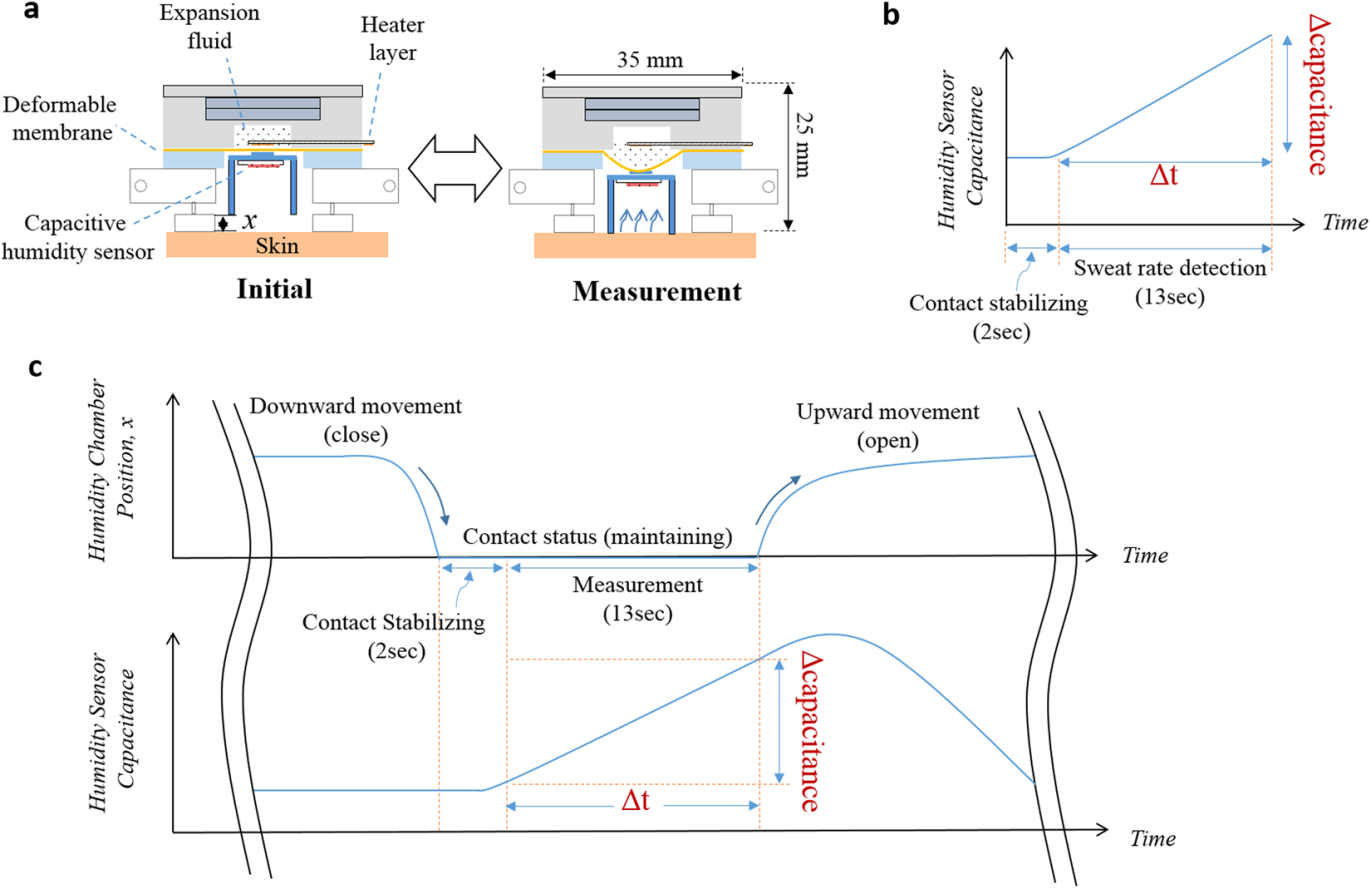
Operation of the watch-type sweat rate sensor: (**a**) operation procedure; (**b**) sweat rate detected by capacitance rising rate; (**c**) humidity chamber movements and capacitance profiles.

Figure 2 shows the working principle of the watch-type sweat rate sensor. The thermo-pneumatic actuator is composed of a battery cover, an expansion fluid cavity, expansion fluid, a heater layer, and a deformable membrane. The heater layer controls the temperature of the expansion fluid. Temperature-dependent vapor pressure of the expansion fluid deforms the deformable membrane, thus moving the humidity chamber upward or downward directions (see Fig. 2a). Relation between the vapor pressure and the temperature is governed by the Clausius-Clapeyron equation^36^.

1 $$P={P}_{boil}{e}^{\frac{{\rm{\Delta }}{H}_{vap}(\frac{1}{{T}_{boil}}-\frac{1}{T})}{R}}$$

where *P* is the vapor pressure of the expansion fluid inside the cavity, *T* is the temperature of the expansion fluid inside the cavity, *P*_*boil*_ is the vapor pressure at boiling temperature, *T*_*boil*_ is the boiling temperature of the expansion fluid, *R* is the gas constant, and *H*_*vap*_ is the enthalpy of vaporization. To optimally minimize the expansion fluid volume, we analyze the time response of the expansion fluid temperature for the volume, through the thermal circuit-based estimation model. Detailed analysis procedure is described in *Methods* section.

Sweat rate is measured by the capacitive humidity sensor located at the upper position inside the humidity chamber. When the humidity chamber is closed by the skin, the capacitive humidity sensor measures the humidity in terms of a capacitance value (see Fig. 2b). The increasing slope in capacitance is the capacitance rising rate, the parameter linearly proportional to the sweat rate^32^. Figure 2c shows the humidity chamber movement and the humidity-dependent capacitance profile. Sweat rate measurement procedure is consist of a skin contact phase and a release phase. During the skin contact phase, the thermo-pneumatic actuator moves the humidity chamber downward. Then, the humidity chamber is in contact with a skin for 15 sec: initial 2 sec is the contact stabilization state and the following 13 sec is the sweat rate detection state. During the release phase, the thermo-pneumatic actuator moves the humidity chamber upward above skin. The collected humidity in the humidity chamber is naturally ventilated through a gap between the chamber and skin. One measurement time including the skin contact phase and the contact release phase takes total 2 min, considering that the previous thermal status monitoring studies^37–39^ monitor the sweat rate every under 3 min.

### Design

Table S1 shows the required specification of the watch-type sweat rate sensors for the design consideration. The watch-type sweat rate sensor is designed to use the input voltage of 6 V by two commercial batteries for moving the humidity chamber (inner diameter: 10 mm, height: 10 mm) with the distance of 3 mm. Total width of the watch-type sweat rate sensor is designed as 35 mm.

The heater material uses a gold due to its high thermal and electrical conductivities. The expansion fluid uses the PF-5060 having low boiling temperature of 56 °C. The deformable membrane uses the commercial 200 µm-thick latex rubber (G5, *Kimberly-Clark*, USA) which is highly flexible (elongation percentage: 826%) with the high tensile strength of 28.5 MPa. The humidity chamber material is PMMA which are durable (Young’s modulus: 1.8~3.1 GPa) and easy to fabricate. The capacitive humidity sensor is the commercially provided micro sensor (SY-HC-1, *SAMYOUNG S&C*, Korea).

**Figure 3:**
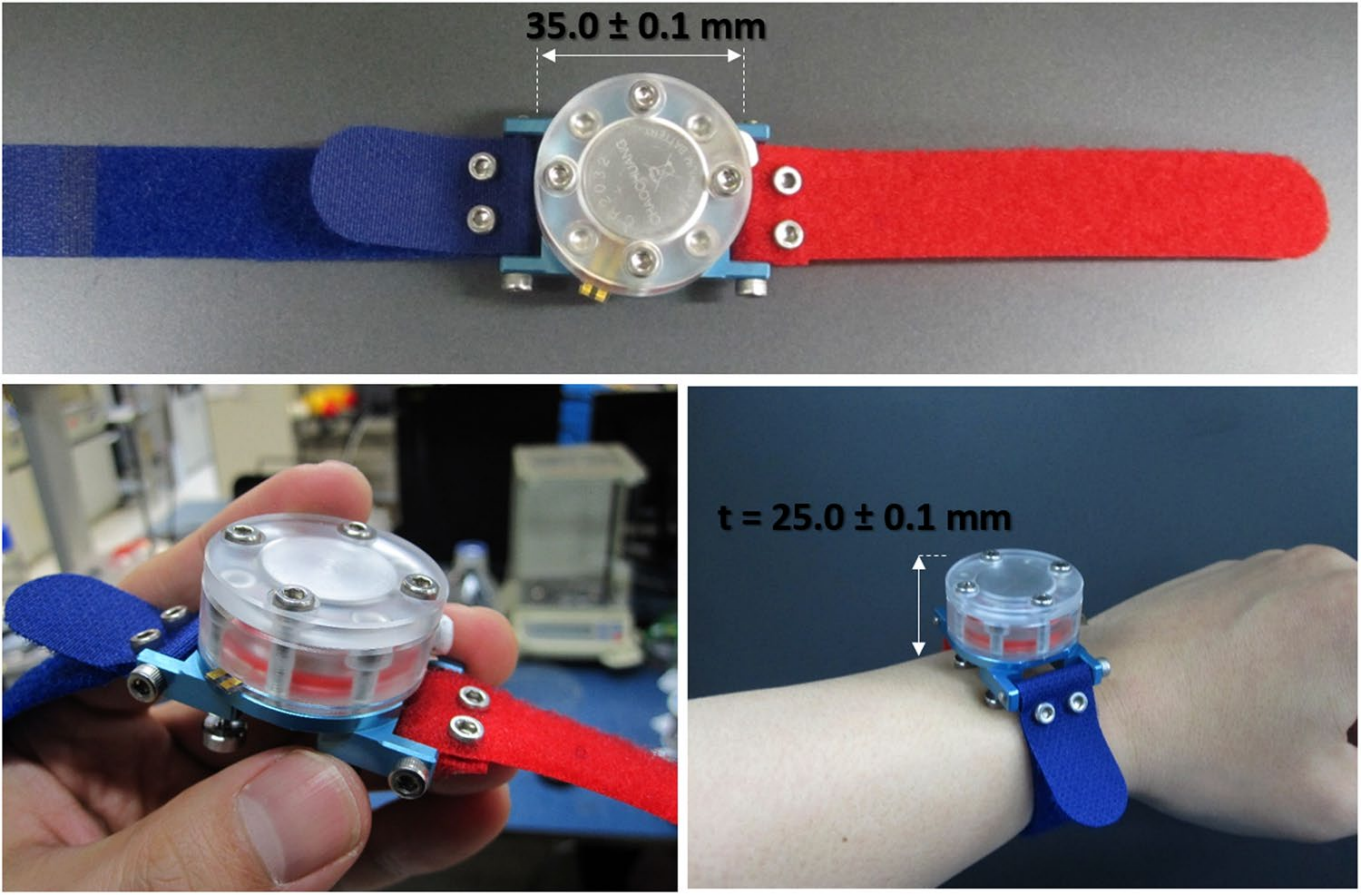
The fabricated watch-type sweat rate sensor.

To decide the expansion fluid volume, we theoretically estimate the arrival time for boiling temperature of the expansion fluid, in the condition of the heater voltage of 6 V. Based on the thermal circuit model, the expansion fluid volume is decided as 0.4 ml in order to move the humidity chamber in 15 sec using 1.8 W (voltage: 6 V, heater resistance: 20 Ω). The detailed procedure for the volume determination is expressed in *Methods* section. The diameter of the deformable membrane is designed as 13 mm, considering humidity chamber’s external diameter of 12 mm. The 0.2 mm-thick deformable membrane can endure the 3 mm deflection. In order to fill the 0.4 ml expansion fluid, the expansion fluid cavity has the 1 mm-diameter hole with a depth of 5 mm. The expansion fluid cavity has another hole at the upper position with the size of 2 mm-dimeter × 4 mm-depth, to mount the two commercial batteries (CR2032) having 2 mm-diameter × 3 mm-thickness. Therefore, total thickness of the expansion fluid cavity is decided as 9 mm. The size of the battery cover is 35 mm-diameter × 3 mm-thickness with the hole size of 2 mm-diameter × 2 mm-depth. In order to minimize the effect of wearable on sweat rate, the present watch-type sweat rate sensor is equipped with the specially designed skin contact legs. The skin contact legs are screw types, thus capable to tune their length up to 5 mm in the range of 3~8 mm. As shown in Fig. 1c, the skin contact legs form constant distance of 1 mm between the sweat rate detector and the skin for natural air ventilation. The width and the height of the heater layer is designed as 22 mm × 5 mm, considering the diameter of the deformable membrane, fabrication yield for 4 inch wafer and the wiring area. The width and the height of the capacitive humidity sensor is 7 mm × 5 mm. The polyimide film of the capacitive humidity sensor is sandwiched by the dual electrodes. The electrodes measure the humidity in terms of the capacitance value which is changed by the humidity-dependent dielectric constant of the polyimide film. The capacitive humidity sensor has the sensitivity of 0.6 pF/% and the operation temperature range of −40~120 °C. The overall size of the watch-type sweat rate sensor is 35 mm-diameter × 25 mm-thickness as shown in Fig. 3.

### Humidity chamber movement characterization

**Figure 4:**
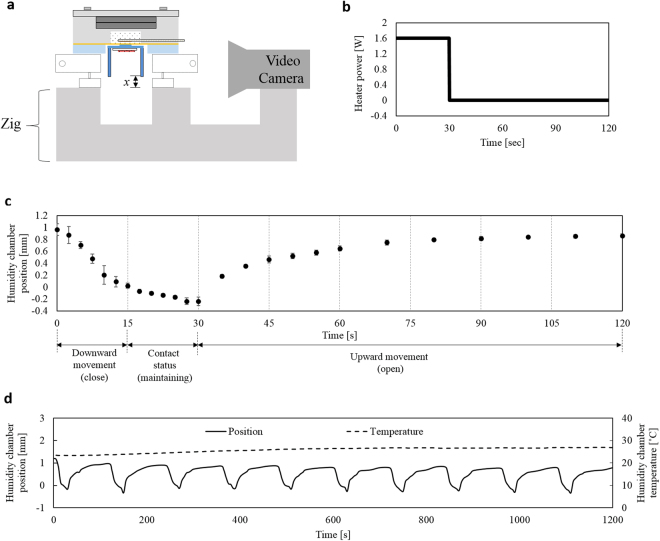
Characterization of the thermo-pneumatic actuation of the humidity chamber: (**a**) experimental setup; (**b**) the input power train, composed of the heater power of 1.6 W for 30 sec and 0 W for 90 sec, for the one cycle humidity chamber movement; (**c**) time-dependent humidity chamber position for the input power train of Fig. 4b; (d) time-dependent humidity chamber position and temperature inside the humidity chamber for the ten cycle of the operation of Fig. 4c.

Figure 4 shows the characterization of the humidity chamber movements by the thermo-pneumatic actuator. Figure 4a shows the experimental setup for the characterization. The watch-type sweat rate sensor is aligned its height with a video camera (SD780IS, *Canon*, Japan) by using a zig. The video camera records the time-dependent humidity chamber position during the actuation. For the chamber movement, we apply the input power train (see Fig. 4b) composed of the heater power of 1.6 W for 30 sec and 0 W for 90 sec for the one cycle humidity chamber movement, where the input voltage is 6 V with a heater resistance of 23 Ω. Figure 4c shows time-dependent humidity chamber position for the input power train of Fig. 4b. 1 mm-movement of the humidity chamber occurs in 15 sec but a tardy movement is shown after 15 sec. The tardy movement is caused by the deformable membrane deflection which decreases a surface level of the expansion fluid. The expansion fluid under the heater layer shows seldom expansion due to a lack of heat transfer from the heater. Therefore we decide the downward movement interval of 0–15 sec and then contact status interval of 15–30 sec. During the contact status, the humidity chamber maintains its position for 15 sec: 2 sec for the contact stabilization; 13 sec for the sweat rate detection. Further movement of the humidity chamber after downward movement is observed as 0.3 mm. Since the elastic human skin can be pressed up to 3 mm^40^, we conclude that the humidity chamber maintains its position for 15 sec. For the chamber upward movement, we turn off the heater voltage and observe the time-dependent position recovery of the chamber. The humidity chamber recovers 85% of its initial position in 80 sec. Recovery time of 80 sec is fast enough for the total measurement time of 2 min. For the repeatability test of the chamber movements, we applied the periodic input power train to the heater for ten times. Figure 4d shows time-dependent humidity chamber position for the ten cycle of the operation of the Fig. 4c. Ten periodic movements of the humidity chamber show the stable movement characteristics with an average movement of (0.86 ± 0.13) mm.

To identify the temperature effect due to expansion fluid during measurement cycle, we also measure the temperature inside the humidity chamber by using a thermocouple attached to the chamber ceiling. As a result, the temperature inside the humidity chamber is measured as under 30 °C (initial and maximum temperatures: 23.4 °C and 26.8 °C) during the humidity chamber movement operations (see dashed line in Fig. 4d). The capacitive humidity sensor has a temperature coefficient of 0.16 pF/°, where the offset change is calculated as 0.5 pF corresponding to humidity change of 0.8% in the range of 23.4~26.8 °C. Since sweat rate is measured by the time rate of the humidity rise (humidity rising rate), this offset change doesn’t affect the sweat rate measurement performance.

### Artificial skin experiment

**Figure 5:**
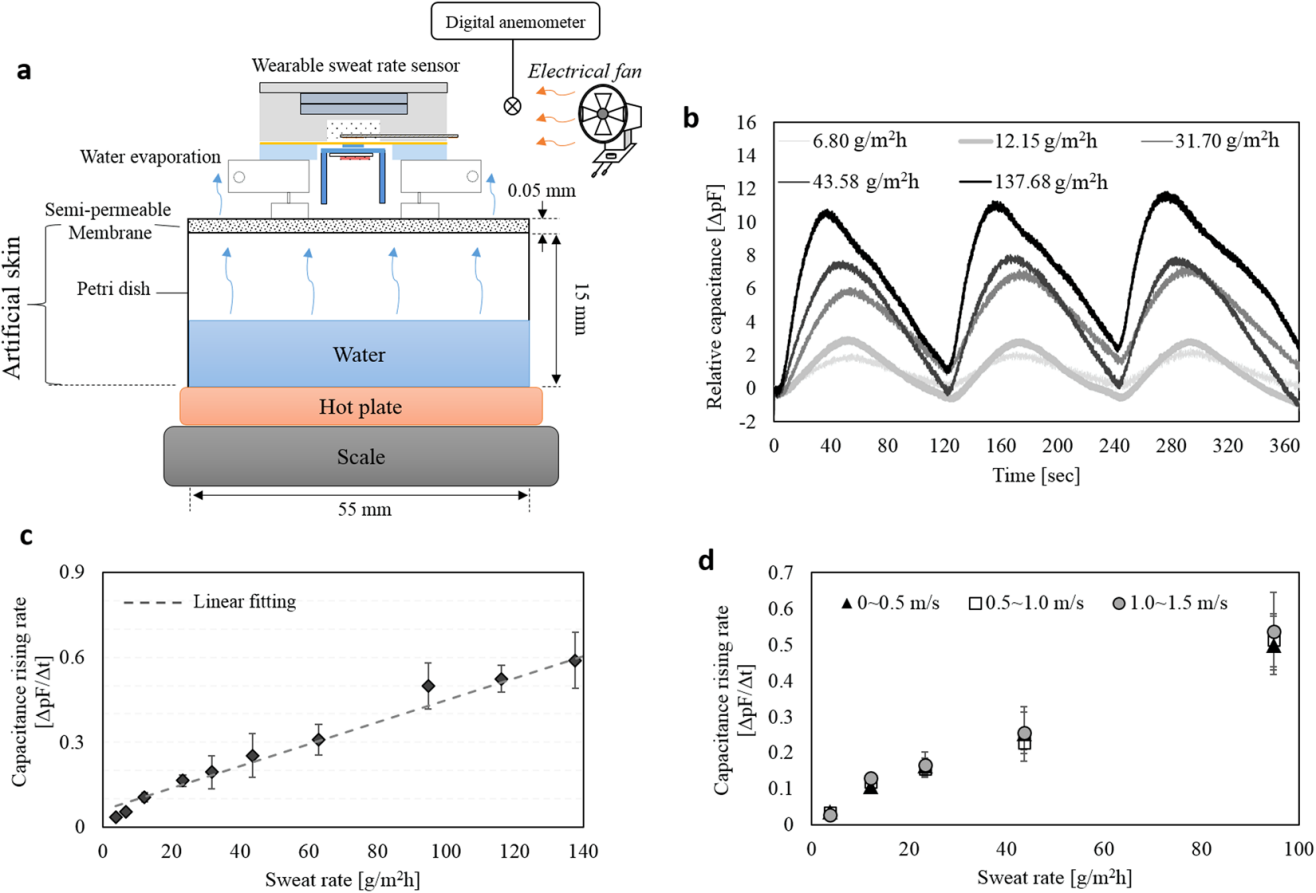
Artificial skin experiment: (**a**) experimental setup for the watch-type sweat rate sensor characterization; (**b**) time-dependent relative capacitance in the five different sweat rate conditions during three measurement cycles; (**c**) capacitance rising rate depending on the sweat rate ranging from 3.76 g/m^2^h to 137.68 g/m^2^h; (**d**) capacitance rising rate depending on the sweat rate, in the three different air velocity conditions in the ranges of 0~0.5 m/s, 0.5~1.0 m/s, and 1.0~1.5 m/s.

The sweat rate measurement experiments are performed under the surrounding temperature and the relative humidity of 25.0 ± 1.0 °C and 50 ± 5%, respectively. Calibration of the sweat rate measurement is conducted by the artificial skin^35^ using wet-cup method^31^ which generates a known sweat rate. Figure 5a shows the structure of the artificial skin. A petri dish filled with water is covered by a semi-permeable membrane (OpSite FlexigridTM, *Smith and Nephew*, England). Water evaporation through the semi-permeable membrane determines the sweat rate. Amount of evaporation is controlled by the layer number of semi-permeable membrane (1, 2 or 4 layers) and the water temperature (25~95 °C) controlled by a hot plate. A scale measures the water mass loss every 10 min for 1 h. Sweat rate is obtained from the water loss mass divided by both the time and the petri dish area. As a result, the artificial skin^31,32,35^ can generate 12 points of sweat rate in the range from 3.76 g/m^2^h to 137.68 g/m^2^h.

We characterize the sweat rate measurement performance of the watch-type sweat rate sensor by using the fabricated artificial skin. The watch-type sweat rate sensor is placed on the semi-permeable membrane of the artificial skin (see Fig. 5a). The periodic input power train (see Fig. 4b) is applied to the heater for the humidity chamber movements (see Fig. 4d). While the humidity chamber periodically moves, LabVIEW records capacitance measured from the capacitive humidity sensor with a sampling rate of 8 Hz. Figure 5b shows relative capacitance depending on time for the five different sweat rate conditions in three measurement cycles. Increasing or decreasing intervals in capacitance correspond to the skin contact phases or the release phases, respectively. At the skin contact phases, greater capacitance rising rate indicates the higher sweat rate. At the release phases, capacitance levels recover their initial position through the natural ventilation. We plot the capacitance rising rate according to the sweat rates. Figure 5c shows capacitance rising rate depending on sweat rate in the artificial skin range. The sweat rate dependent capacitance rising rate shows a sensitivity of 0.039 (pF/s)/(g/m^2^h), a linearity of 97.9%, an uncertainty of 13.01 g/m^2^h, and a limit of detection of 27.6 g/m^2^h. The watch-type sweat rate sensor takes 2 min for the each sweat rate measurement. The previous paper^41^ reported that the sweat rate change under moderate heat stress takes 30 min for determining the human thermal status. The other previous paper^42^ reported that the small sweat rate change of 60 g/m^2^h takes 45 min for the person with a normal thermal status. Therefore, the measurement period of 2 min is enough fast to be used for the monitoring applications under the indoor environments or in the normal activities.

To characterize the wind-resistant performance of the watch-type sweat rate sensor, we observe the capacitance rising rate for five different sweat rates under the air velocity range up to 1.5 m/s, corresponding to the average human walking speed^43,44^. An electrical fan controls the air flow on the basis of the digital anemometer near the watch-type sweat rate sensor (see Fig. 5a). Figure 5d shows capacitance rising rate depending on sweat rate, exposed by the three different air velocity ranges of 0~0.5 m/s, 0.5~1.0 m/s, and 1.0~1.5 m/s. As a results, sweat rate dependent capacitance rising rate shows a sensitivity of 0.0049 (pF/s)/(g/m^2^h), 0.0050 (pF/s)/(g/m^2^h), and 0.0053 (pF/s)/(g/m^2^h) and a linearity of 99.4%, 98.6% and 98.8%, under the air velocity range of 0~0.5 m/s, 0.5~1.0 m/s, and 1.0~1.5 m/s, respectively. Maximum differences of the sensitivity and the linearity show 8.2% and 0.7%, respectively. Therefore, the wind in the range of the human walking speed have little effect on the sweat rate measurement of the watch-type sweat rate sensor.

The watch-type sweat rate sensor consumes average power of 0.4 W: 1.6 W for 30 sec and 0 W for 90 sec, every 2 min for the thermo-pneumatic actuation. Using the two commercial batteries (CR2032, 3.0 V, 240 mAh), it is possible to operate the watch-type sweat rate sensor for about 4~5 hours when the measurement period is 2~3 min. Total weight of the watch-type sweat rate sensor including two batteries is measured as (30 ± 1) g.

### Human experiment

**Figure 6:**
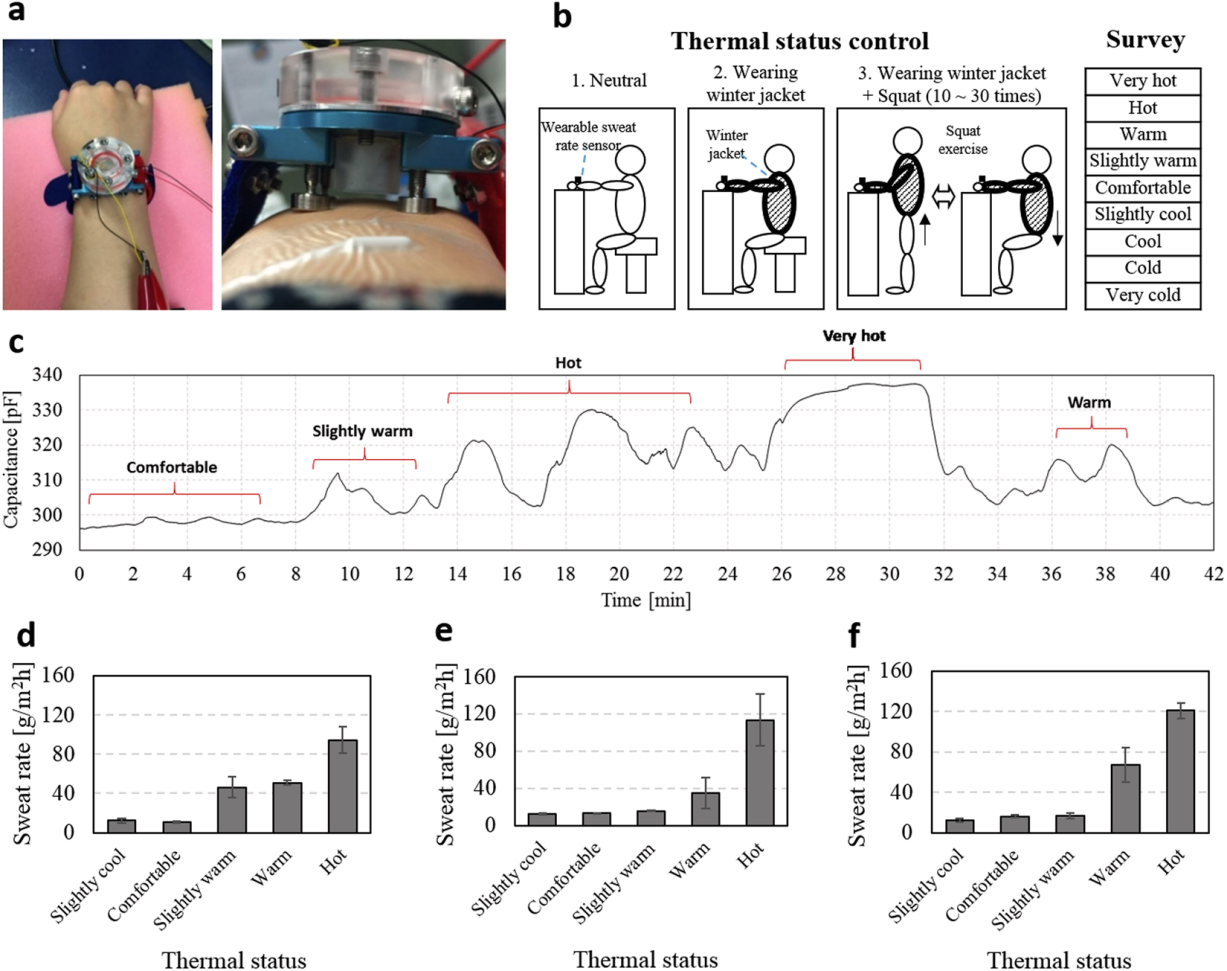
Human experiment: (**a**) watch-type sweat rate sensor in the wrist with 1 mm gap between the human skin and the humidity chamber; (**b**) the thermal status control using three different conditions and the survey used for thermal status evaluation; (**c**) typical profile of capacitance depending on time, where the capacitance is measured by the humidity sensor from subject 1 during the thermal status control; (**d**–**f**) sweat rate depending on thermal status for the three different subjects (subject 1–3).

We measure the human sweat rate by using the watch-type sweat rate sensor to analysis the sweat rate differences in the various human thermal statuses. KAIST IRB (Institutional Review Board) approves the present human experiments (approval ID: KH2011-18). Before the experiments, all subjects receive detailed explanation of the human experiments and sign the informed consents. Three healthy subjects with the average age of 27.7 ± 3.2 are recruited. Environment conditions of air temperature, relative humidity, black globe temperature and air velocity are 25 °C ± 1 °C, 45% ± 5%, 24.5 °C ± 0.5 °C, and 0~0.5 m/s, respectively. Each subject sit comfortably, placing his or her right arm on a desk. The watch-type sweat rate sensor is placed on the right arm as shown in Fig. 6a. The screw-type skin contact legs adjust their length to make the 1 mm-gap between the humidity chamber and right arm skin. In order to control the individual thermal status, we design three conditions (see Fig. 6b): 1) neutral, 2) wearing winter jacket, and 3) wearing winter jacket with squat exercise (10~30 times). During the thermal status control, we measure subject’s sweat rate with an interval of 2 min while the subject is asked to check the individual thermal status guided by the questionnaire^45^. The questionnaire is composed of ‘very cold’, ‘cold’, ‘cool’, ‘slightly cool’, ‘comfortable’, ‘slightly warm’, ‘warm’, ‘hot’, and ‘very hot’. These nine statuses are designated as −4, −3, −2, −1, 0, +1, +2, +3, and +4, respectively (see Table S2). We measure at least three sweat rates for each thermal status, calculating average sweat rates and the standard deviations. Figure 6c shows typical profile of capacitance depending on time, where the capacitance is measured by the humidity sensor from subject 1 during the thermal status control. In the thermal status range from 0 to +3, the capacitance rising rate increases as the subject feels hotter. In the thermal status of +4, however, capacitance is saturated and the capacitance rising rate cannot be detectable because the speed of the sweat rate generation is faster than that of the ventilation speed. Increment of the gap between the humidity chamber and the skin can be a potential solution to solve this saturation phenomenon. Figure 6d–f show sweat rate depending on thermal status for the subjects 1–3, respectively. In the case of subject 1, the sweat rate difference between 0 and +1, +1 and +2, and +2 and +3 are 35.01 g/m^2^h, 4.76 g/m^2^h, and 43.44 g/m^2^h, respectively. In the case of subject 2, the sweat rate difference between 0 and +1, +1 and +2, and +2 and +3 are 2.46 g/m^2^h, 19.21 g/m^2^h, and 78.60 g/m^2^h, respectively. Average sweat rate difference is calculated as (33.42 ± 40.01) g/m^2^h. In the case of subject 3, the sweat rate difference between 0 and +1, +1 and +2, and +2 and +3 are 0.45 g/m^2^h, 50.74 g/m^2^h, and 53.84 g/m^2^h, respectively. Average sweat rate difference is calculated as (35.01 ± 29.97) g/m^2^h. Average sweat rate difference is calculated as (27.74 ± 20.34) g/m^2^h. As a result of three subjects experiment, the difference in sweat rate between each thermal status is average 32.06 ± 27.19 g/m^2^h. Therefore, the watch-type sweat rate sensor experimentally calculates the sweat rate changes for each thermal status in the range from 0 to +3.

Average sweat rates for the four different thermal statuses of 0, +1, +2, and +3 are measured as (13.40 ± 2.48) g/m^2^h, (26.04 ± 17.33) g/m^2^h, (50.95 ± 16.17) g/m^2^h and (109.57 ± 13.80) g/m^2^h, respectively, in three subjects measurements. Inter-subject variation of the sweat rates are expressed by Coefficient of Variation (CV) which is defined as the ratio of the standard deviation to the average sweat rate. Inter-subject variation of the sweat rates for the four different thermal statuses of 0, +1, +2, and +3 are calculated as 18.5%, 31.7%, 66.5% and 12.6%, respectively; average inter-subject variation is calculated as 32.3%. This sweat rate variation from person to person is because of the personal differences such as life environment, heredity, constitution, healthy status, etc. To normalize the sweat rate variation of over 30%, an individualization process is required through the long-term data acquisition and the database establishment.

## Discussion and Conclusion

Scientific contribution of the present study is to improve the practical applicability of the sweat rate sensor by reducing its operation power and weight. The artificial skin experiment as well as the human experiment prove that the present sweat rate sensor shows high applicability for a wearable use. Previous portable prototype^33^ is difficult to use in the wearable applications due to high operation power of 12.5 W. The present device makes it possible to implement the principle into wearable monitoring watch. The present device has 4 hours of the operation time with two commercial batteries, by reducing its operation power to 1.4 W. The key method used in this device is the development of the heat circuit model capable to not only minimize the expansion fluid volume from 8 ml to 0.4 ml, but also reduce the power consumption from 12.5 W to 0.4 W.

In our knowledge, the state of art includes a few wearable types of sweat rate monitoring systems^29,30,33,46,47^. However, these systems are not dealing with the ventilation error by wind or water accumulation, thus not subject to comparison with the present watch-type sweat rate sensor having automatic ventilation function. The conventional wearable sweat rate monitoring systems with ventilation^21–23^ have the bulky sizes with the weights of over 1 kg due to their extra ventilation devices, 300 times heavier than the present device. Therefore, these systems are not applicable to wearable applications. When we consider the commercial watch-type wearable electronic devices, Galaxy Gear S3 and Apple Watch Series 3 (Aluminum - 42 mm) have the weights of 64.0 g and 32.3 g, respectively. Therefore, the 30 g-weight of the present watch-type sweat rate sensor is comparable to those of commercial wearable electronic devices.

In conclusion, we present the wind-resistant, low-weight and low-power watch-type sweat rate sensor and experimentally verify its performance and applicability. Table 1 shows the specification of the watch-type sweat rate sensor. A low-power miniaturized thermo-pneumatic actuator is integrated for the automatic natural ventilation. The integrated thermo-pneumatic actuator uses PF-5060 (boiling temperature: 56 °C) as an expansion fluid and optimally minimizes the expansion fluid volume to 0.4 ml. The total weight of the watch-type sweat rate sensor is 3.0% of that of the previous force ventilation sweat rate sensors. The watch-type sweat rate sensor reduces the operation power to 12.8% and the total weight to 47.6% compared to the previous natural ventilation sweat rate sensors. Total size of the watch-type sweat rate sensor is 35 mm-diameter × 25 mm-thickness with the total weight of 30 g. The thermo-pneumatic actuator using the operating voltage of 6 V moves the humidity chamber 0.86 mm ± 0.13 mm with the movement period of 2 min. The watch-type sweat rate sensor is capable to measure the sweat rate with a linearity of 97.9% and a sensitivity of 0.0039 (pF/s)/(g/m^2^h) over a range of 3.76 to 137.68 g/m^2^h. The sweat rate measurement sensitivity changes less than 10% under the air velocity of up to 1.5 m/s corresponding to human walking speed, thus independent to the wind. The human experiment reveals that sweat rate difference for each thermal status measured in three subjects is calculated as average (32.06 ± 27.19) g/m^2^h in the range of four stages of thermal status including ‘comfortable’, ‘slightly warm’, ‘warm’, and ‘hot’, thus experimentally demonstrating the sweat rate change according to the human thermal status. The present watch-type sweat rate sensor is light and insensitive to the wind, thus having high potential for the mobile human thermal comfort monitoring applications, such as cognitive air-conditioning systems or smart monitoring jackets.

**Table 1:** Specification of the watch-type sweat rate sensors.

Specification		Measured performance
Sweat rate detection	Range	3.67~137.68 g/m^2^h
	Sensitivity	0.0039 (pF/s)/(g/m^2^h)
	Linearity	97.9%
	Measurement period	2 min
Humidity chamber movement		0.86 ( ± 0.13) mm
Input voltage		6 V
Average consuming power per measurement period (2 min)		0.4 W
Size		Overall diameter × total thickness: (35 ± 0.5) mm × (25 ± 0.5) mm (including batteries*)
Weight		30 ( ± 1.0) g (including two batteries*)
Wind-resistant range		~1.5 m/s (human walking speed**)

^*^CR2032, lithium coin, 3 V, 240mAh.

**A. Pepin, *et al*. *Spinal Cord* (2003).

## Methods

### Theoretical analysis for the expansion fluid volume determination

The simplified thermodynamic model of Figure S1 is used for determining the expansion fluid volume (*v*). The model of Figure S1 can be analogized by electrical circuit model of Figure S2a, composed of the input electrical power, *Q*; the thermal resistance of *R*_1_, *R*_21_, and *R*_22_; the thermal capacitance of the expansion fluid, *C*_1_. The equivalent circuit elements are defined as follow:

2-1 $$Q=\frac{{V}^{2}}{R}$$

2-2abc $${R}_{1}=\frac{1}{{h}_{1}{A}_{1}},{R}_{21}=\frac{1}{{h}_{2}{A}_{2}},{R}_{22}=\frac{{L}_{2}}{{k}_{2}{A}_{2}}$$

2-3 $${C}_{1}=mc=vdc$$

where, *Q*, *V*, and *R* are the heat transfer rate from the heater into the expansion fluid, the electrical voltage applied to the heater, and the electrical resistance of the heater; *h*_1_ and *h*_2_ are the convection heat transfer coefficient of the expiation fluid and the surrounding air; *k*_2_ and *L*_2_ are the conduction heat transfer coefficients and the thickness of the expansion fluid cavity; *A*_1_ and *A*_2_ are the areas of the heater and the deformable membrane; *m*, *v*, *d*, and *c* are the mass, volume, density, and the heat capacity of the expansion fluid, respectively. Circuit model of Figure S2a can be simplified as Figure S2b. From the Figure S2b, we obtain a governing equation of heat transfer:

2-4a $$Q=\frac{{T}_{0}-{T}_{1}}{{R}_{1}}={C}_{1}\frac{dT}{dt}+\frac{{T}_{1}-{T}_{surr}}{{R}_{2}}$$

where,

2-4b $${R}_{2}=\frac{1}{\frac{1}{{R}_{21}}+\frac{1}{{R}_{22}}}=\frac{{R}_{21}{R}_{22}}{{R}_{21}+{R}_{22}}$$

where *T*_0_ and *T*_*surr*_ are the temperature of the heater layer and the surrounding air, respectively. The first term of the right side of Eq. (2-4a) represents the thermal energy used to increase the temperature of the expansion fluid in the expansion fluid cavity. The second term of Eq. (2-4a) indicates the heat dissipated through the wall of the expansion fluid cavity and the surrounding air. The solution of the differential equation of Eq. (2-4) is obtained as

2-5 $${T}_{1}-{T}_{surr}=Q{R}_{2}(1-{e}^{-\frac{t}{{R}_{2}{C}_{1}}})$$

By substituting Eq. (2-4b) into Eq. (2-5), we find the equation for the temperature of the expansion fluid, *T*_1_ depending on the heat power, *Q*.

2-6 $${T}_{1}={T}_{surr}+Q{R}_{2}(1-{e}^{-\frac{t}{{R}_{2}{C}_{1}}})$$

where,

$$\,{R}_{2}=\frac{1}{\frac{1}{{R}_{21}}+\frac{1}{{R}_{22}}}=\frac{{R}_{21}{R}_{22}}{{R}_{21}+{R}_{22}}$$

By using Eq. (2-6), we theoretically estimate the time response of expansion fluid temperature for the various expansion fluid volume. Figure S3 shows the estimated time response of the expansion fluid temperature for the various expansion fluid volume of 0.2 ml, 0.4 ml, 0.6 ml and 1.0 ml. Equation (2-6) uses the values of the electrical voltage applied to the heater (*V*), the electrical resistance of the heater (*R*), the surrounding temperature (*T*_*surr*_), the *R*_21_, the *R*_22_, the *C*_1_, the area of deformable membrane (*A*), the heat transfer coefficient of air (*h*), the thermal conductivity of PMMA (*k*), the PMMA length (*L*), the specific heat capacity of PF-5060 (*c*), and the density of PF-5060 (*d*) are 6 V, 50 Ω, 25 °C, 94.2 K/W, 84.8 K/W, 1.4 J/K, 5.31 × 10^−4^ m^2^, 20 W/(m^2^·K), 0.2 W/(m·K), 0.009 m, 1.05 J/(g·K), and 1.68 g/ml, respectively. Larger expansion fluid volume is capable of the large movement of the humidity chamber, whereas, it takes longer time for movement. Therefore, we determine the optimal expansion fluid volume as 0.4 ml, which takes under 15 sec for reaching boiling temperature of 56 °C.

### Fabrication

Figure S4 shows the fabrication process of the watch-type sweat rate sensor. The heater layer fabrication (Fig. S4a) commences with a 4 inch, 500 μm-thickness glass wafer. After the glass wafer cleaning by dipping in a piranha solution (H_2_SO_4_:H_2_O_2_ = 1:1) for 10 min and rinsing in DI water, Cr/Au of 100 Å/1000 Å is deposited on the wafer surface by evaporation. The deposited Cr/Au film is patterned by the conventional photolithography technique. The Cr/Au patterned glass wafer is diced into each heater layer element by a dicing saw. The expansion fluid cavity is fabricated (Fig. S4b) by PMMA machining. The deformable membrane (Fig. S4c) is the commercialized latex rubber which thickness is 0.2 mm. The skin contact legs layer is fabricated (Fig. S4d) by Al machining. After fabricating the elements, we assemble and joint all the prepared elements (Fig. S4e) using screws. Finally, the expansion fluid of PF-5060 is injected into the expansion fluid cavity by a syringe to complete the fabrication of the present watch-type sweat rate sensor (Fig. S4f). The fabricated watch-type sweat rate sensor has a size of (35 ± 0.5) mm-diameter × (25 ± 0.5) mm-thickness with a total weight of (30 ± 1) g.

### Sweat rate calibration using wet-cup method

The artificial skin (Fig. 5a) use “wet-cup method” to calibrate the sweat rate sensing ability, which is a general calibration method^24,31,32,48^. The wet-cup method provides quantitative sweat rate with a unit of g/m^2^h, thus considered as reference methods to sweat rate detection.

Wet-cup method is composed of petri-dish filled with water and covered by a semipermeable membrane. Control of the water evaporation rate through the membrane is accomplished by tuning the water temperature and the semipermeable membrane thickness. Then, the water evaporation (sweat rate) is calculated by the three measured data:

3 $$SR[g/{m}^{2}h]=\frac{W[g]}{A[{m}^{2}]\cdot T[h]}$$

where, *SR* is the sweat rate, *W* is the weight of water loss, *A* is the area of petri dish, and *T* is the time. This calculated sweat rate value is used for the sweat rate calibration when the sensor is placed on the semipermeable membrane. The artificial skin (wet-cup method) offers a reference to characterize the performance of the sweat rate sensor.

### Human experiment

KAIST Institutional Review Board (IRB) has approved the present human experiments. All sweat rate experiments performed on human subjects were carried out with informed consent under the guidelines and regulations of the KAIST IRB, ID number KH2011-18.

## Electronic supplementary material

Supplementary information for the research methods
