# Supplementary material for: Wearable Sweat Rate Sensors for Human Thermal Comfort Monitoring
Source: Sci Rep. 2018 Jan 19;8:1181. doi: 10.1038/s41598-018-19239-8 (PMC5775419; doi:10.1038/s41598-018-19239-8)
Supplement: Supplementary file 1 — Supplementary information for the research methods [file 41598_2018_19239_MOESM1_ESM.pdf]

Wearable Sweat Rate Sensors for Human Thermal Comfort Monitoring

Jai Kyoung Sim[1], Sunghyun Yoon[1] & Young-Ho Cho[1,*]

1. Department of Bio and Brain Engineering, Korea Advanced Institute of Science and Technology (KAIST), 271 Daehak-ro, Yuseong-gu, Daejeon 34141, Republic of Korea.

*Correspondence and requests for materials should be addressed to Y.-H.Cho (email: nanosys@kaist.ac.kr)

Table S1. Required specification of the watch-type sweat rate sensor

Design and specification	Value	Remark
Sweat rate detection range	> 110 g/m^{2}h	Human sweat rate in strong thermal stress: 110 g/m^{2}h *
Measurement period	< 3 min	Time resolution for sweat rate change: 3 min**
Humidity chamber movement	3.0 mm	85% ventilation time < 1min
Input voltage	< 6 V	Two commercialized batteries***
Average consuming power	< 1.0 W	Average power consumption of portable device: 1.0~1.2 W ****
Width	40 ~ 60 mm	< Human wrist width
Total weight	< 100 g	Considering device portability
Wind-resistant range	~ 1.5 m/s	Human walking speed: 1.5 m/s*****

***** K. Blazejczyk, *et. al.*, *Geogr. Pol.* (2013).

****** Caleb H., *et. al.*, *Clin. Auton. Res.* (2001).

******* CR2032, lithium coin, 3V, 240mAh

**** A. Pepin, *et. al.*, *Spinal Cord* (2003).

Table S2. Questionnaire* for thermal status evaluation

Time [min]	Thermal status								
	-4 (Very cold)	-3 (Cold)	-2 (Cool)	-1 (Slightly cool)	0 (Comfort-able)	1 (Slightly warm)	2 (Warm)	3 (Hot)	4 (Very hot)
2									
4									
6									
8									
10									
12									
14									
16									
18									
20									
22									
24									
26									
28									
30									
32									
34									
36									
38									
40									
42									
44									
48									
50									

*F. Dominik, *et. al.*, *Theor. Appl. Climatol*. (2016)

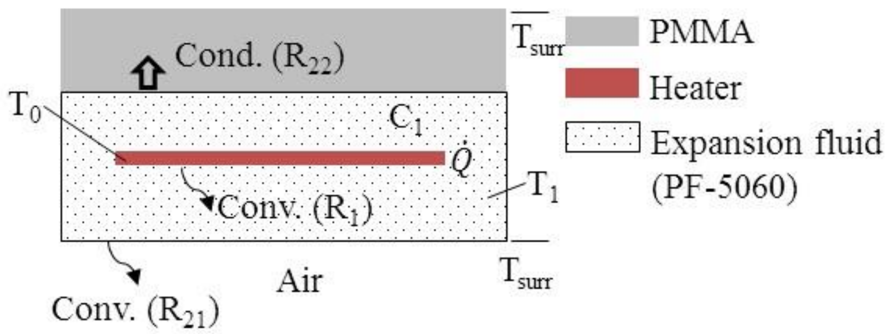

Figure S1. Simple heat transfer model of the thermo-pneumatic actuator.

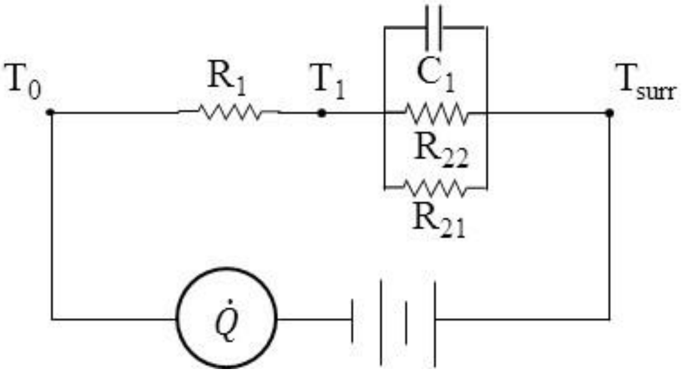

(a)

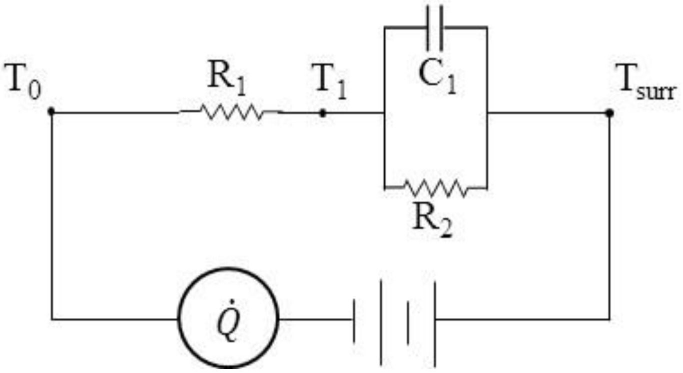

(b)

Figure S2. Electrical circuit analogy of Figure S1:

(a) equivalent electrical model; (b) simplified electrical model.

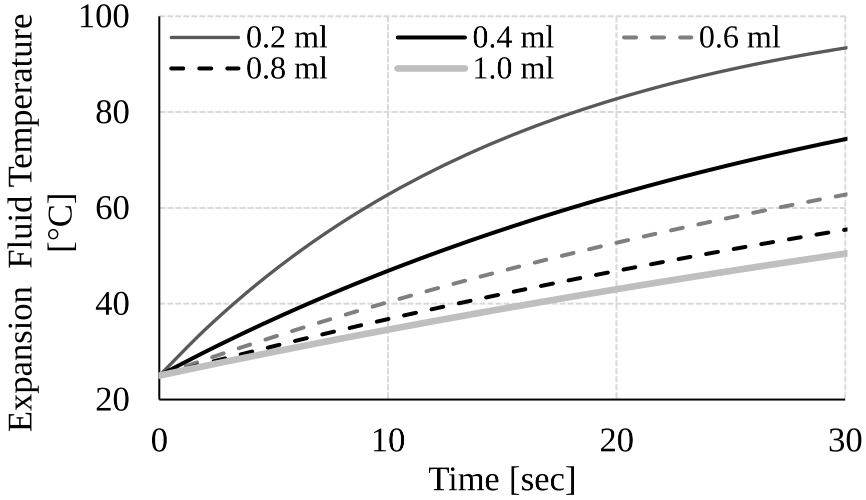

Figure S3. Estimated expansion fluid temperature depending on time for the five different expansion fluid volumes of 0.2 ml, 0.4 ml, 0.6 ml, 0.8 ml and 1.0 ml, where the heater power is 1.8 W.

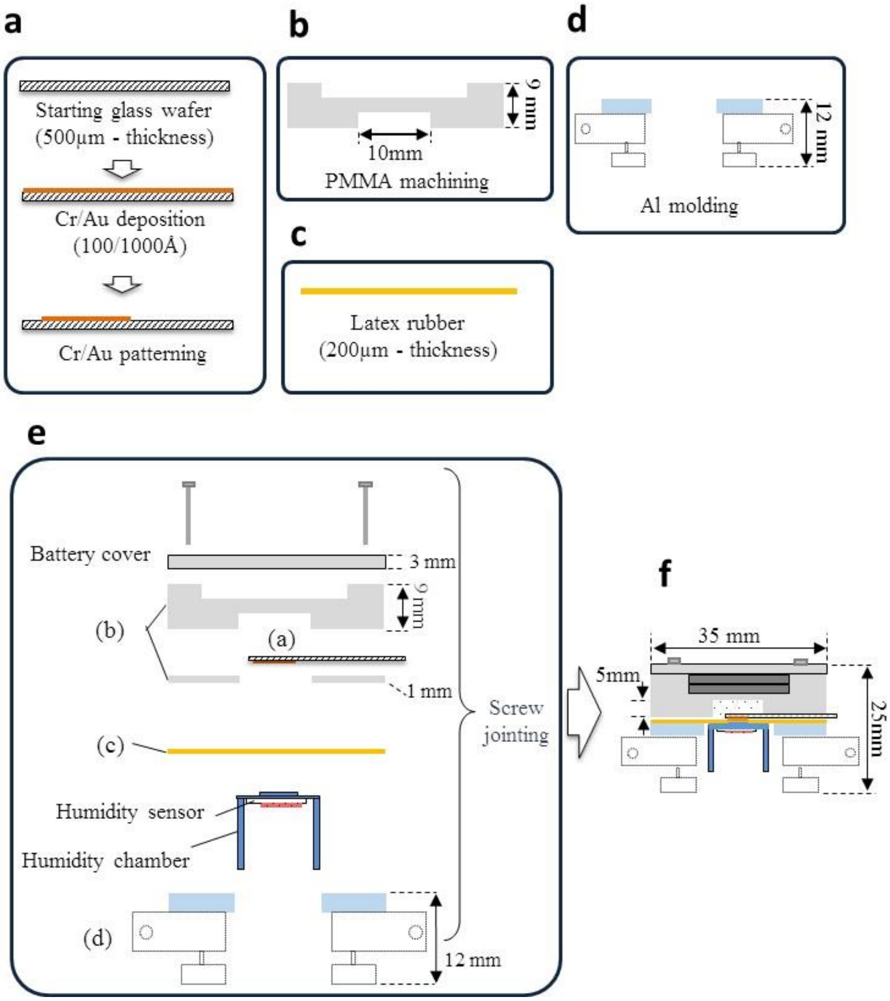

Figure S4. Fabrication process of the watch-type sweat rate sensor: (a) heater layer; (b) expansion fluid cavity; (c) deformable membrane; (d) skin contact legs; (e) assembly and bonding; (f) completed device.
